# Salinity and high pH reduce denitrification rates by inhibiting denitrifying gene abundance in a saline-alkali soil

**DOI:** 10.1038/s41598-023-29311-7

**Published:** 2023-02-07

**Authors:** Yongchun Pan, Dongli She, Zhenqi Shi, Taohong Cao, Yongqiu Xia, Jun Shan

**Affiliations:** 1 College of Agricultural Science and Engineering, Hohai University, Nanjing, 210098 China; 2 Jiangsu Province Engineering Research Center for Agricultural Soil-Water Efficient Utilization, Carbon Sequestration and Emission Reduction, Nanjing, 210098 China; 3 Key Laboratory of Soil and Sustainable Agriculture, Changshu National Agro-Ecosystem Observation and Research Station, Institute of Soil Science, Chinese Academy of Sciences, Nanjing, 210008 China

**Keywords:** Biogeochemistry, Environmental sciences

## Abstract

Denitrification, as the main nitrogen (N) removal process in farmland drainage ditches in coastal areas, is significantly affected by saline-alkali conditions. To elucidate the effects of saline-alkali conditions on denitrification, incubation experiments with five salt and salt-alkali gradients and three nitrogen addition levels were conducted in a saline-alkali soil followed by determination of denitrification rates and the associated functional genes (i.e., *nirK*/*nirS* and *nosZ* Clade I) via N_2_/Ar technique in combination with *q*PCR. The results showed that denitrification rates were significantly decreased by 23.83–50.08%, 20.64–57.31% and 6.12–54.61% with salt gradient increasing from 1 to 3‰, 8‰, and 15‰ under 0.05‰, 0.10‰ and 0.15‰ urea addition conditions, respectively. Similarly, denitrification rates were significantly decreased by 44.57–63.24% with an increase of the salt-alkali gradient from 0.5 to 8‰. The abundance of *nosZ* decreased sharply in the saline condition, while a high salt level significantly decreased the abundance of *nirK* and *nirS*. In addition, the increase of nitrogen concentration attenuated the reduction of *nirK*, *nirS* and *nosZ* gene abundance. Partial least squares regression (PLSR) models demonstrated that salinity, dissolved oxygen (DO) in the overlying water, N concentration, and denitrifying gene abundance were key determinants of the denitrification rate in the saline environment, while pH was an additional determinant in the saline-alkali environment. Taken together, our results suggest that salinity and high pH levels decreased the denitrification rates by significantly inhibiting the abundance of the denitrifying genes *nirK*, *nirS,* and *nosZ*, whereas increasing nitrogen concentration could alleviate this effect. Our study provides helpful information on better understanding of reactive N removal and fertilizer application in the coastal areas.

## Introduction

Nitrogen (N) is an essential nutrient for crop growth, thus synthetic N fertilizers are commonly applied in the farmland to achieve high yield. However, yield increases that rely on increased N inputs likely result in a high risk of reactive N (Nr) losses^1^. Excess N fertilizer application in farmlands leads to substantial Nr releasing into rivers and lakes through runoff and drainage, which results in a series of environmental problems including agricultural non-point source pollution^2–4^. The drainage ditch system is a type of wetland in the agricultural ecosystem between the output of farmland field and the receiving water bodies (i.e., lakes and rivers). The migration and transformation of pollutants in these drainage ditches plays a vital role in controlling their final output load^5^. The urgency of eliminating Nr from farmland drainage ditches and reducing the environmental pollution from exogenous N in rivers and lakes has drawn substantial scientific and public attention. Denitrification which is the microbial reduction of nitrate/nitrite (NO_3_^−^/NO_2_^−^) to the gaseous products nitric oxide (NO), nitrous oxide (N_2_O), and dinitrogen (N_2_) under hypoxic conditions^6^, occurs essentially in all water-flooded systems and is considered the major N removal process in ditch systems^7–10^.

Mitigating agricultural non-point source N pollution is quite important in agricultural production areas since N fertilizer is easily lost therein. Saline-alkali soil in the coastal region of eastern China has become an important land resource for agriculture with increasing population growth and socioeconomic development^11–14^. However, due to its special physicochemical properties, the N fertilizer use efficiency of crops grown therein is generally very low, even to 5.63%^15^ and below the national average of 34.3%^16^ increasing the risk of Nr loss. Meanwhile, the salt and alkali in farmland soils can flow into the farmland drainage ditches with runoff and drainage, resulting in an increase of salt and alkali contents in these ditches. Salinization and alkalization can alter the fundamental physicochemical nature of the soil–water environment, not only in ionic concentrations but also in chemical equilibria and microbiological composition^17,18^. The increased ionic concentrations change physicochemical processes and chemical equilibria, facilitate aggregation and sedimentation of suspended solids, and induce the fast displacement of cations [e.g., ammonium (NH_4_^+^)] that are bound to the cation adsorption complex in the sediment^18^. Moreover, the microbial community structure and diversity of denitrifying bacteria in the soils are greatly affected by salinity^19^. Soil salinity adversely affects the activity of soil enzymes^14^ and the abundances of N cycling associated genes^20,21^. The altered physicochemical and biological properties induced by salinization and alkalization will inevitably influence N transformation processes therein. For instance, salinization can directly affect denitrification rate by increasing or decreasing N availability in the soil^22,23^. Some studies have also shown that the denitrifying community structure changes greatly under high salinity conditions and sometimes even loses its function^24,25^. Moreover, the high pH value of saline-alkali soil is another crucial factor affecting the composition of the denitrifying bacterial community^26^, enzyme activity^27^, and the abundance of associated functional genes^6^.

Saline-alkali soil has a high salinity and pH^28^; however, previous studies on denitrification and denitrifying bacteria in saline-alkali soil were mostly restricted to low salinity soils^19,20^ and rarely involved alkaline environments^27^. It has been reported that high saline-alkali environments may induce physiological stress in denitrifying bacteria and can eventually result in large shifts in the composition of denitrifying bacteria and their associated ecosystem functions^17^. Furthermore, saline-alkali soil with a high salinity and pH often results in large losses of farmland fertilizer into the ditches, increasing the input of exogenous N into the ditches. A comprehensive understanding of how salt and alkali contents regulate denitrification by affecting the composition of denitrifying bacteria is critical to improve the removal of Nr in farmland drainage ditches and to reduce non-point source pollution in coastal areas. However, until recently, relatively little was known about the interaction effects of high saline-alkali conditions and exogenous N on the denitrification rate and the composition of denitrifying bacteria in farmland drainage ditches of coastal areas. To analyze the effect of saline-alkali condition on denitrifying bacteria, we focused on process-specific functional genes, mainly *nirK*, *nirS*, and *nosZ*, which encode pivotal reductases associated with each nitrate reduction pathway^21^ and are frequently used as functional markers for analyzing denitrifying microbe communities^29^. In this study, we examined the variation in denitrification rates of three urea levels under five salt and salt-alkali gradients (from low to high) through a flooded incubation experiment and investigated how the abundance of three denitrifying functional genes changed in saline and saline-alkali environments. Concurrently, we determined the controlling factors of the denitrification process using a partial least squares regression (PLSR) model and explored the key determinants of Nr removal in the drainage ditches of farmlands with saline-alkali soils. Our study addressed the question: what are the key determinants of denitrification in ditch systems under saline-alkali conditions and the underlying microbial mechanisms?

## Materials and methods

### Soils

The experimental soils were collected from a ditch in Liuzong Village (32°12′N, 120°42′E) in the town of Juegang, Rudong Country, Jiangsu Province. This area is a typical coastal reclamation area and is mainly used for agriculture. The sampled soils were transported to the laboratory, salt-leached, air-dried, ground and sieved to 5 mm. The soil had a silt loam texture with a pH of 8.0, a soil electrical conductivity (EC_1:5_) of 2.24 mS cm^−1^, a cation exchange capacity (CEC) of 4.99 cmol·kg^-1^, and an organic carbon content (OC) of 3.1 g kg^−1^. Additional details on the general chemistry of this soil can be found in Pan et al.^30^.

### Experimental design

The incubation experiment was conducted in the Water-Saving Park of Hohai University (Nanjing, 31°57′N, 118°50′E, 144 m above sea level) from October 2020 to November 2020 to investigate the effects of different salt and salt-alkali gradients on denitrification rates under three N addition levels. Different salt (sodium chloride, NaCl)^30^ and salt-alkali (sodium bicarbonate, NaHCO_3_)^31^ gradients were set, including a control treatment (CK, without salt and alkali addition), four salt addition treatments (S1, S2, S3 and S4: 1‰, 3‰, 8‰ and 15‰ of soil mass, respectively) and four salt-alkali addition treatments (A1, A2, A3 and A4: 0.5‰, 1‰, 3‰ and 8‰ of soil mass, respectively). The exogenous N treatments included three analytically pure urea addition levels (N1, N2 and N3: 0.05, 0.10 and 0.15 g kg^−1^ soil, respectively). Each treatment was replicated three times.

Different concentrations of NaCl and NaHCO_3_ solutions were gently sprayed onto the sieved soil samples to prevent soil agglomeration, and sprayed and mixed several times to prevent an influence of uneven salt distribution on the results. Soil samples were placed into incubators (PVC, 5 mm thickness, 340 × 270 × 130 mm internal size) after being naturally dried, and each incubator was filled with 8.0 kg soil. Details on procedure of the incubation are provided in previous studies^30^. The incubation was maintained in a shallow water layer of approximately 5 cm for two weeks to stabilize the soil properties and restore microbial communities. Three urea concentration solutions were subsequently applied to the corresponding incubator. An undisturbed sediment sampler was used to collect the overlying water–sediment samples after 24 h of fertilization.

### Denitrification rate measurements

The denitrification (DNF) rates were measured using membrane inlet mass spectrometry (MIMS, Bay Instruments, Easton, MD, USA) according to the protocol by Kana et al.^32^, which quantifies changes in dissolved N_2_:A_r_ ratios within the water overlying the sediments. Details on this procedure are provided in previous studies^6,33^ and Supplementary Fig. S1. Nitrogen removal efficiency (RE) is the ratio of the nitrogen removed by denitrification within 24 h to the nitrogen added in the incubator. More details on the calculation of Nitrogen removal efficiency are shown in Supplementary Text S1.

### Environmental factor measurements

After 24 h of fertilization, before measuring the denitrification rates, overlying water samples (100 mL each) and sediment samples (0–5 cm layer, approximately 20 g for each) were collected to measure environmental parameters, including dissolved oxygen (DO) content, EC, pH, sodium ion (Na^+^) and inorganic nitrogen concentration of the overlying water–sediment system. The NH_4_^+^-N and NO_3_^–^N of the filtered water and soil extracts were analyzed using a flow injection analyzer (Skalar Analytical, Breda, The Netherlands). More details on these environmental factor measurements are provided in a previous study^30^ and the summary of the results showed in Supplementary Table S1.

### DNA extraction and quantification of denitrifying gene abundance

The sediment samples were immediately collected from the surface layer of the overlying water–sediment interface at a depth of 5 mm for cryopreservation to quantitative analysis of denitrifying gene abundance after the denitrification rates measurement. Total genomic deoxyribonucleic acid (DNA) samples were extracted from frozen sediment subsamples using an Ultra Clean Soil DNA Isolation kit (MoBio Laboratory, Carlsbad, CA, USA) according to the manufacturer’s instructions. For PCR amplification of all functional genes, a microfluidics Fluidigm Gene Expression chip was used to quantify all genes simultaneously^34^. The gene-specific primers are listed in Table 1, and more details are shown in Supplementary Text S2.

**Table 1:** Primers for amplification of denitrifying genes in this study.

Genes	Primer ID	Sequence (5′–3′)
*nirK*^35^	nirK-876C	ATYGGCGGVCAYGGCGA
	nirK-1040	GCCTCGATCAGRTTRTGG
*nirS*^36^	nirS-Cd3af	GTNAAYGTNAARGARACNGG
	nirS-R3cd	GASTTCGGRTGSGTCTTGA
*nosZ* Clade I^37^	nosZ-2F	CGCRACGGCAASAAGGTSMSSGT
	nosZ-2R	CAKRTGCAKSGCRTGGCAGAA

### Statistical analysis

One-way analysis of variance (ANOVA) was carried out and examined with Duncan’s test to investigate the statistical significance of the impact of different salt or salt-alkali gradients on denitrification rates and gene abundances under the same urea level. Variations in the data for denitrification rates and gene abundance affected by the interactions of salt or salt-alkali and N were evaluated by two-way ANOVA. The normality of all data was checked and met before the ANOVA was performed. Pearson’s correlation analysis was conducted to determine the relationship between the denitrification rate, environmental parameters and denitrifying gene abundance. Statistical significance was determined at a probability level of 0.05. Redundancy analysis (RDA) was performed with salinity, pH, DO and N concentration as variables to identify the primary environmental factors affecting the abundance of denitrifying genes. A classical regression method was not ideal for this dataset because some variables may be highly correlated, which could lead to reduced statistical power of the classical regression model and even cause a misinterpretation of regression coefficients. A partial least squares regression (PLSR) model which allows for a strong collinearity between the variables was used to examine the effect of the variables on the denitrification rate^38^. All the above statistical analyses were performed using SPSS 22.0 (SPSS IBM., Armonk, NY, USA). All figures were created using Origin 9.0 (OriginLab Corporation, USA).

## Results

### Variation in denitrification rates

**Figure 1:**
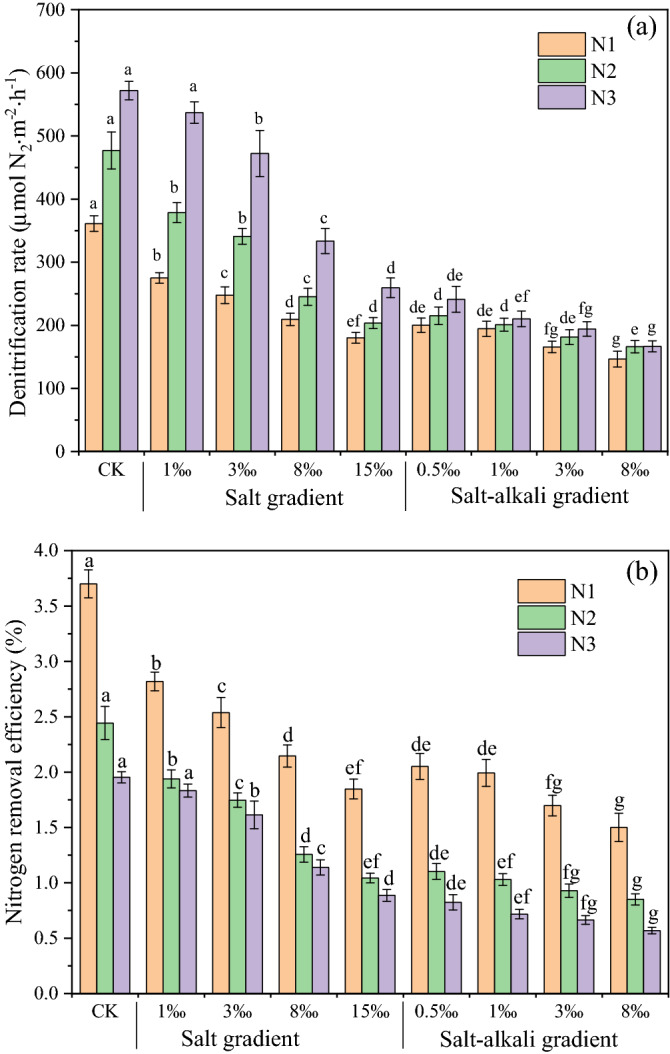
Denitrification rates (**a**) and nitrogen removal efficiency caused by denitrification (**b**) under various salt/salt-alkali gradients and urea levels within 24 h. The different letters above the bars indicate significant differences between the treatments within salt/salt-alkali gradients under the same urea level. Note: N1, N2, and N3 represent 0.05‰, 0.10‰ and 0.15‰ urea addition, respectively. Bars represent denitrification rates; error bars denote standard deviations of three independent repetitions (n = 3). Different letters indicate the significant differences among treatments under the same nitrogen condition (p < 0.05, one-way ANOVA followed by Duncan’s test).

The denitrification rates of the salt and salt-alkali treatments were 180.33–536.93 μmol N_2_ m^−2^ h^−1^ and 146.51–241.13 μmol N_2_ m^−2^ h^−1^, respectively (Fig. 1a). Both salt and salt-alkali treatments significantly affected denitrification rates under the same urea level, and the denitrification rates decreased with increasing salt and salt-alkali addition. Compared with the CK treatment, the denitrification rates decreased rapidly under the salt-alkali treatments and were much lower than those under the salt treatments. An increase in urea significantly increased the denitrification rates (Fig. 1a). Two-way ANOVA showed that the interactions between urea and salt/salt-alkali treatments significantly (*p* < 0.01) affected the denitrification rates (Table 2).

**Table 2:** *P* values and F values from two-way ANOVA of urea levels (N), salt gradients (S)/salt-alkali gradients (A) and their interaction (N × S/N × A) on denitrifying gene abundances and denitrification rates.

Source	df	*nirK*		*nirS*		*nosZ* Clade I		Denitrification rate	
		*P*	F	*P*	F	*P*	F	*P*	F
N^a^	2	0.00**	10.97	0.00**	12.59	0.00**	6.50	0.00**	324.59
S	4	0.00**	52.86	0.00**	19.17	0.00**	69.22	0.00**	190.38
N × S	8	0.10	1.91	0.31	1.25	0.76	0.62	0.00**	11.63
N^b^	2	0.00**	14.82	0.10	3.67	0.00**	14.76	0.00**	49.11
A	4	0.00**	52.30	0.00**	60.97	0.00**	94.90	0.00**	475.57
N × A	8	0.14	1.69	0.97	0.29	0.04*	2.43	0.00**	17.06

N^a^ and N^b^ represent the effect of urea on the dependent variables when interacting with salt and alkali, respectively. Significance levels: *P < 0.05, **P < 0.01.

With increased salt or salt-alkali addition, the N removal efficiency within 24 h of denitrification decreased gradually (Fig. 1b). For the salt treatments, the N removal efficiency of N1 was significantly higher than that of N2 and N3. Moreover, there was no significant difference in N removal efficiency between the N2 and N3 levels under the same salt conditions. However, under salt-alkali conditions, the N removal efficiency of the N2 level treatments was significantly higher than that of the N3 level (Fig. 1b).

### Response of denitrifying gene abundance to salt and salt-alkali gradients

**Figure 2:**
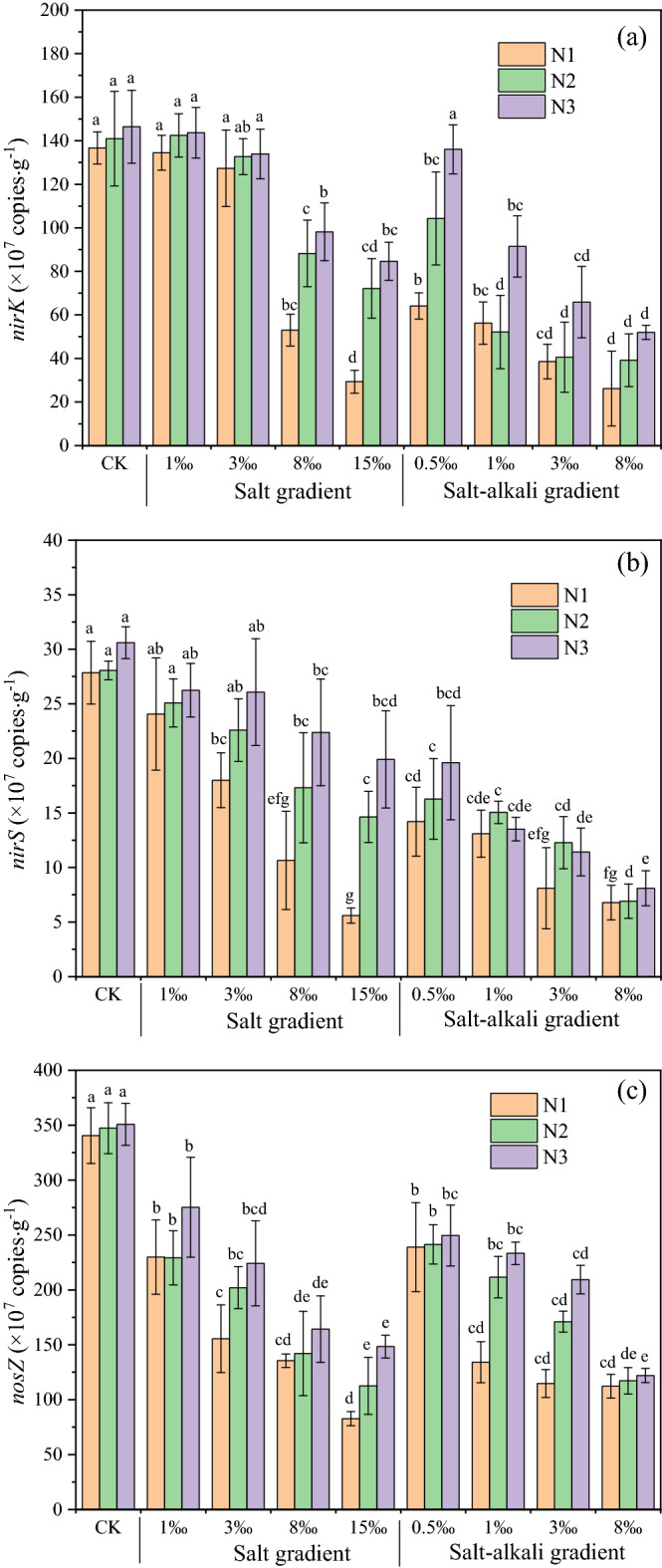
Abundance of the denitrifying functional genes *nirK* (**a**), *nirS* (**b**), and *nosZ* (**c**) under various salt/salt-alkali gradients and urea levels. The different letters above the bars indicate significant differences between the treatments within salt/salt-alkali gradients under the same urea level. Note: N1, N2, and N3 represent 0.05‰, 0.10‰ and 0.15‰ urea addition, respectively. Bars represent abundance of the denitrifying functional genes; error bars denote standard deviations of three independent repetitions (n = 3). Different letters indicate the significant differences among treatments under the same nitrogen condition (p < 0.05, one-way ANOVA followed by Duncan’s test).

Two-way ANOVA showed that there was no significant interactive effect between urea and salt, nor between urea and salt-alkali content, on the denitrifying gene abundance (Table 2). Therefore, one-way ANOVA was used to test whether salt and salt-alkali treatments had significant impacts on denitrifying gene abundance under each urea level. For the salt treatments, the abundances of the *nirK*, *nirS* and *nosZ* genes were between 2.93 × 10^8^–1.44 × 10^9^ copies g^−1^, 5.60 × 10^7^–2.62 × 10^8^ copies g^−1^ and 8.28 × 10^8^−2.75 × 10^9^ copies g^−1^ of dry sediment, respectively (Fig. 2a–c). The abundance of all three denitrifying functional genes decreased with increasing salt addition. Salt and urea levels had no significant effect on the abundance of the *nirK* gene at salt additions of 1‰ and 3‰, while at 1‰ salt additions, salt and urea levels had no significant effect on *nirS* gene abundance. When the salt gradient was greater than 3‰, the abundance of the *nirK* and *nirS* genes decreased significantly, and the urea level had a significant and positive impact on the *nirK* and *nirS* gene abundance (Fig. 2a,b). Compared to *nirK* and *nirS*, *nosZ* gene abundance was more sensitive to salt and decreased significantly when salt increased slightly (Fig. 2c).

For the salt-alkali treatments, the abundances of the *nirK*, *nirS* and *nosZ* genes were within the range of 2.62 × 10^8^–1.36 × 10^9^ copies g^-1^, 6.78 × 10^7^–1.96 × 10^8^ copies g^−1^ and 1.12 × 10^9^–2.49 × 10^9^ copies g^−1^ of dry sediment, respectively (Fig. 2a–c), much less than those of the salt treatments with the same content. The increase in urea slowed the decreasing trend of *nirK* gene abundance caused by the salt-alkali treatment, and when the salt-alkali addition was greater than 3‰, urea had no significant effect on the *nirK* gene abundance (Fig. 2a). The urea level had no significant effect on the *nirS* gene abundance under salt-alkali conditions (Fig. 2b). For the *nosZ* gene, the gene abundance of N1 treatment decreased rapidly when the salt-alkali gradient increased, while that of N3 treatment decreased gradually. The decreased range of gene abundance of N2 treatment was between N1 and N3 treatment (Fig. 2c).

### Relationship between denitrifying gene abundance and environmental factors

**Figure 3:**
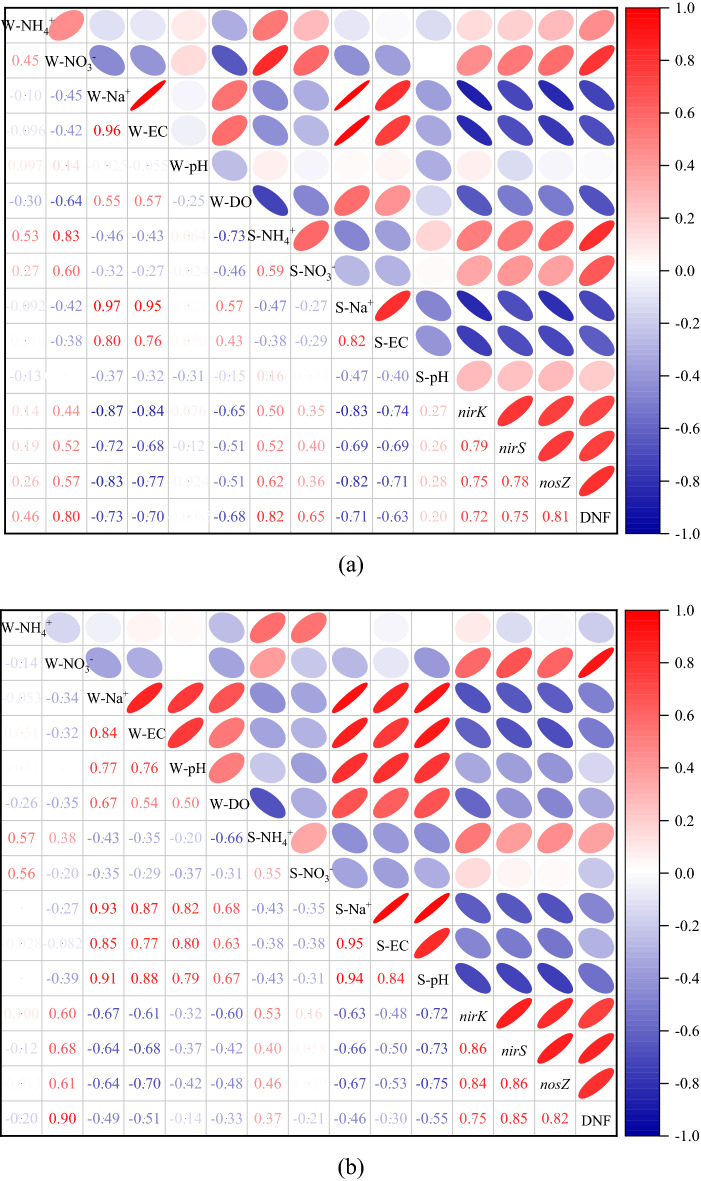
Heatmap of the correlation among the denitrification rates, denitrifying gene abundances and environmental factors. (**a**) Saline environment; (**b**) Saline-alkali environment. DNF indicates the denitrification rate. The numbers on the lower left indicate the correlation coefficients of the denitrification rates, denitrifying gene abundances and environmental factors; a coefficient value > 0.31 indicates that the correlation between the two variables is significant at a 95% confidence level. The shaded ellipses on the upper right indicate the direction and magnitude of the interactions between two variables. The darker the red/blue shades are, the greater the positive/negative correlations between two variables. “W-” and “S-” indicate the overlying water and sediment, respectively. The figures were created using Origin 9.0 (OriginLab Corporation, USA).

Pearson’s correlation analysis was performed to evaluate the correlation between denitrification rates and denitrifying gene abundance. The denitrification rates were significantly correlated with the abundance of the denitrifying genes *nirK*, *nirS* and *nosZ* under both saline and saline-alkali conditions (Fig. 3a,b).

Regardless of saline and saline-alkali conditions, the denitrification rates were positively correlated with N concentrations, including NH_4_^+^-N and NO_3_^–^N, but negatively correlated with variables related to salinity, including Na^+^ and EC, of the overlying water and sediment (Fig. 3a,b). Moreover, the denitrification rates were also negatively correlated with the DO of the overlying water. In the saline conditions, the pH of the overlying water and sediment did not change significantly when the salt gradient increased. However, the denitrification rates were negatively and significantly correlated with the pH of the sediment in the saline-alkali conditions (Fig. 3b). The pH of the overlying water–sediment system played different roles in the denitrification rates in the saline and saline-alkali conditions. The denitrification rates decreased with the increase of pH in the saline-alkali conditions, while pH had no significant effect on denitrification rates in the saline conditions.

**Figure 4:**
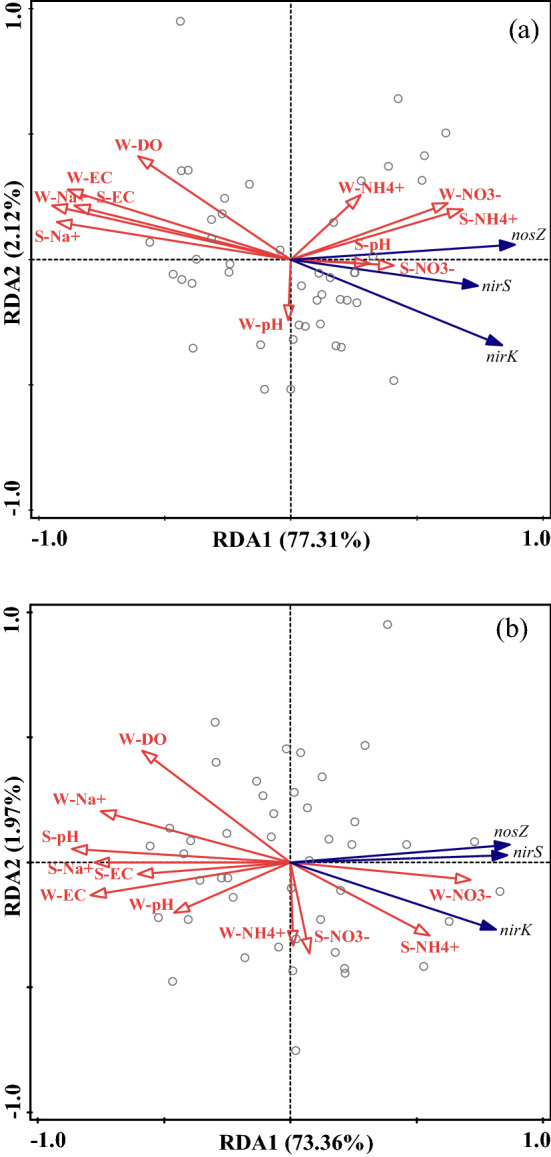
Redundancy analysis (RDA) plot of the relationship between the abundances of denitrifying functional genes and environmental factors (**a**, saline environment; **b**, saline-alkali environment). Solid blue lines indicate the denitrifying gene abundances, and solid red lines show environmental factors. “W-” and “S-” indicate the overlying water and sediment, respectively.

Similar to the relationship between the denitrification rates and environmental factors, the denitrifying gene abundance was negatively correlated with salinity and DO in both the saline and saline-alkali conditions (Fig. 3a,b). Furthermore, in the saline-alkali condition, the abundances of the *nirK*, *nirS* and *nosZ* genes were significantly and negatively correlated with pH. Redundancy analysis (RDA) was performed to further evaluate the relationship among environmental factors and the abundance of denitrifying genes (Fig. 4). The RDA showed that the physico-chemical properties of the overlying water–sediment system explained 79.43% (RDA1, 77.31%; RDA2, 2.12%) and 75.33% (RDA1, 73.36%; RDA2, 1.97%) of the variance in the denitrifying gene abundance in the saline and saline-alkali conditions, respectively. As Fig. 4 shows, the denitrifying gene abundances were positively correlated with N concentration and negatively correlated with salinity and DO. Meanwhile, Fig. 4b indicates that the denitrifying gene abundances were also negatively correlated with the pH of the overlying water and sediment in the saline-alkali condition.

### The determinants of denitrification in saline-alkali soils

To investigate the effect of environmental factors on denitrification rates and denitrifying gene abundances in more detail, we input those variables in a PLSR model. In this model, salinity, pH, DO, N concentrations and denitrifying gene abundances were considered predictors and denitrification rates were considered responding variables. We established two PLSR models based on salt and salt-alkali conditions according to the different the overlying water–sediment system environments. The two models explained 90.2% and 91.8% of the variance in the denitrification rates in the saline and saline-alkali environments, respectively (Tables S2, S3).

**Figure 5:**
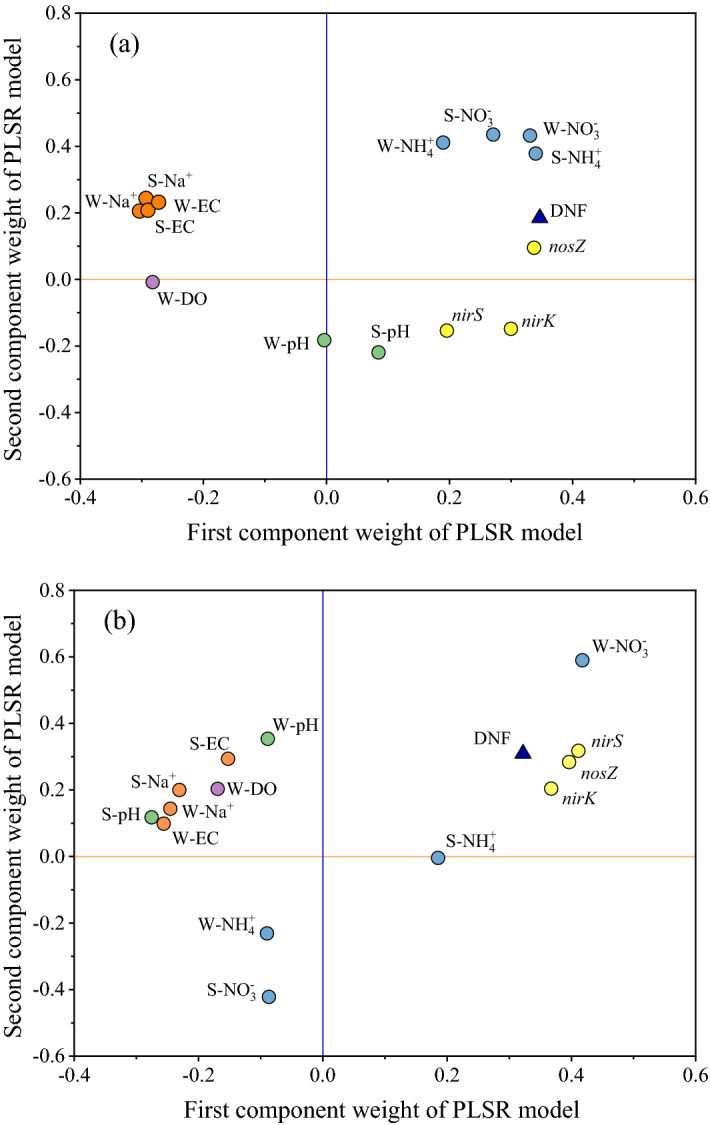
Weight plots of the first and second PLSR components for denitrification rates (**a**, saline environment; **b**, saline-alkali environment). DNF indicates the denitrification rate. “W-” and “S-” indicate the overlying water and sediment, respectively.

The relationship among environmental factors found by the regression analysis could be seen in the PLSR weight plot of component 1 versus component 2 (Fig. 5). For the saline environment, component 1 accounted for 50.7% of the variation in the independent variables (environmental factors) and 83.4% of the variation in the denitrification rates. Component 2 accounted for another 15.1% and 6.8%, resulting in a total of 65.8% and 90.2%, respectively (Table S2, Fig. 5a). The DO of the overlying water and the system salinity were clustered along component 1, and the negative relationship between these factors and the denitrification rates was obvious since they were found at opposite ends of component 1 (Fig. 5a). For the saline-alkali environment, components 1 and 2 accounted for 53.0% and 18.3% of the variation in the environmental factors and 67.7% and 24.1% of the variation in the denitrification rates, respectively (Table S3, Fig. 5b). Denitrification rates were negatively correlated with the overlying water DO, salinity and pH of the system because they were located at both ends of component 1. In contrast, NO_3_^–^N in the overlying water, NH_4_^+^-N in the sediment and the abundance of denitrifying genes positively correlated with the denitrification rates since they were in the same region as the denitrification rates (Fig. 5b).

**Figure 6:**
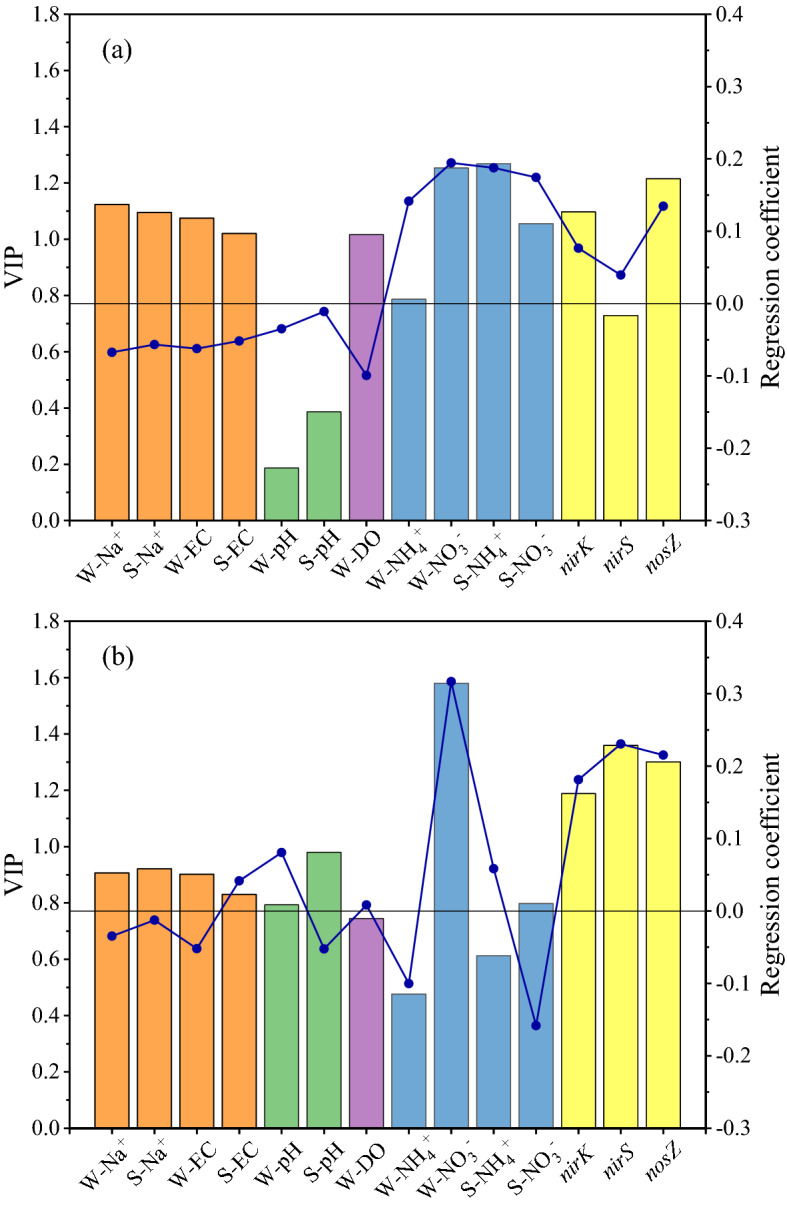
Variable importance for the projections (bars) and regression coefficients (lines) for each predictor of denitrification rates in a saline environment (**a**) and a saline-alkali environment (**b**). VIP, variable importance in prediction. “W-” and “S-” indicate the overlying water and sediment, respectively.

**Figure 7:**
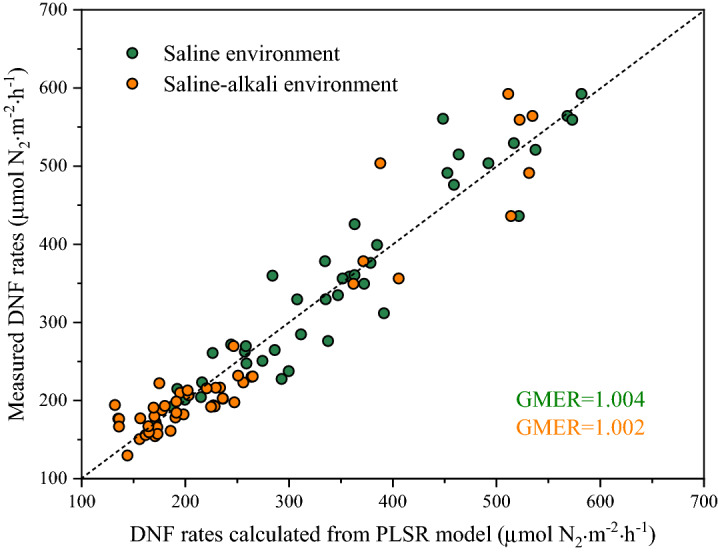
Measured versus calculated denitrification rates based on the PLSR model in the saline and saline-alkali environments. The dotted black line represents a 1:1 line.

Variable importance in prediction (VIP) generated by PLSR analysis reflects the contribution of the independent variables to the denitrification rates; that is, the greater the VIP value is, the greater the explanatory ability of the environmental factors on the denitrification rates (Fig. 6). It is generally considered that independent variables with VIP values > 1.0 are important, independent variables with VIP values between 0.5 and 1.0 are of secondary importance, and independent variables with VIP values less than 0.5 are not important. In the saline environments, the salinity (EC and Na^+^ concentration), DO, N concentration (NO_3_^–^N in the overlying water, NO_3_^–^N and NH_4_^+^-N in the sediment) and denitrifying gene abundance (*nirK* and *nosZ*) were identified as important predictors with VIP values > 1 (Fig. 6a). The VIP value of pH was < 1, which indicated that it was not an important indicator of the saline environment in the PLSR model (Fig. 6a). In the saline-alkali environment, NO_3_^–^N in the overlying water and the abundance of denitrifying genes (*nirK*, *nirS* and *nosZ*) were important predictors in the PLSR model, and salinity, DO, pH and N concentration (NO_3_^–^N and NH_4_^+^-N in the sediment) were the second most important predictors (Fig. 6b). In contrast to the saline environment, the pH and salinity showed similarly important contributions to the denitrification rates in the PLSR model of the saline-alkali environment (Fig. 6b). The geometric mean error ratio (GMER), which is calculated from the error ratio of measured vs. predicted values, is widely used in the literature to describe model biases. A GMER equal to 1 corresponds to an exact match between measured and predicted data, and therefore, the best model will have a GMER close to 1^39^. The GMER of the two PLSR models that we established in the saline and saline-alkali environments were 1.004 and 1.002, respectively (Fig. 7).

## Discussion

### Effects of saline-alkali environments on denitrification

Coastal ecosystems are hotspots for N cycling^21^, especially for removal of Nr from farmland drainage ditches through denitrification process^5^. Salinity plays a critical role in the denitrification process of drainage ditch ecosystem. Elevated salinity has been reported to alter N concentration^21^, the substrate for denitrification, and decrease denitrification significantly (Fig. 1a). The results from this study have shown that the shifts in substrate availability and salinity were largely responsible for the variation in denitrification rates, and salinity also affected the availability of the substrate. High Na^+^ concentrations cause rapid NH_4_^+^ mobilization from the cation exchange sites of sediment in the short term^18^ and increase the NH_4_^+^ availability in soil porewater^23^, which promotes the volatilization of ammonia^30,40^ and reduces the substrate in the process of nitrification and denitrification^41^. Additionally, the decreased of NO_3_^–^N concentration in the overlying water with the increase of salinity directly reducing the availability of denitrification substrate (Table S1).

Denitrification rates are generally considered to increase with increasing soil pH in acidic soils^42,43^, and this result was obtained in the saline environment in the present study, although this correlation was not significant (Fig. 3a). However, the denitrification rates were significantly and negatively related to sediment pH in the saline-alkali environment, and the pH of the sediment was far greater than that in the saline environment. Compared with the saline environment, the denitrification rates of the saline-alkali environment decreased even more (Fig. 1a), and besides salinity and DO (Fig. 6a), high pH was also a restrictive factor for denitrification in the saline-alkali soil (Fig. 6b). The presence of large amounts of alkaline compounds, such as bicarbonate ion (HCO_3_^−^), in the saline-alkali environment, which hydrolyze to produce hydroxide ions (OH^−^), causing an increase in system pH. The NO_3_^–^N concentration in the overlying water in the saline-alkali environment with high pH was further reduced, which decreasing the availability of denitrification substrate (Table S1). In summary, salinity and high pH negatively affected denitrification by reducing the availability of N in the short term. However, with the increase of salt-alkali gradients, the denitrification continued to decrease, while the availability of nitrate nitrogen was not decrease significantly. This indicated that there were other reasons that inhibited the denitrification rates, which will be discussed in the next section.

### Microbial mechanisms behinds salt and alkali

The denitrification rate is essentially a nitrate reduction process mediated by microbial functional groups. In many cases, the denitrifying microorganisms are controlled by environmental factors and gradually adapts to the environment^44^. This study provides a detailed experimental comparison of denitrifying gene abundance in different saline and saline-alkali environments. Although this study is based on manipulated sediments recovered from dry soils, the restored microbial communities originally came from field soils and therefore represented in situ conditions to some extent^21^. While there is limited information on the relationship between salinity and community structure, this study provided strong evidence that high salinity concentrations significantly and negatively affect the gene abundances of denitrifiers. Dominant genera are substituted by salt-adaptable microbes as salinity increases^45^. A decline in the abundance of denitrifying genes was found with increasing salt or salt-alkali conditions (Fig. 2), which is similar to the findings of previous research^21,46,47^. However, salinity exhibited a limited influence on *nirK* and *nirS* abundances in this study, and low salinity concentrations had no significant effect on the abundance of *nirK* and *nirS* (Fig. 2a,b). The abundance of *nosZ* gene, encoding reductase for the last step of denitrification, was more sensitive to salinity than *nirK* and *nirS*, and played a dominant role in denitrification under low salt conditions as evidenced by the concurrent decrease of denitrification rates and *nosZ* gene abundances (Fig. 2c). As salinity continued to rise, the abundances of *nirK* and *nirS* decreased significantly. Nevertheless, the relative importance of *nirK* and *nirS* for denitrification in saline environments remains controversial^20,21,48^. A study in the San Francisco Bay estuary revealed that salinity had a significant negative correlation with *nirK* abundance and a significant positive correlation with *nirS* abundance^49^. Another study in a Jiulong River estuary mudflat revealed that an increase in salinity inhibited the abundances of *nirK* and *nosZ*, while *nirS* abundances remained stable^21^.

The identification of salinity as a strong driver of denitrifying gene abundance is consistent with several studies^21,44,50^. Moreover, salinity is a key regulator that decreases denitrification in coastal ecosystems, primarily as a result of its effect on denitrifiers^51^. There are several mechanisms by which salinity affects microbial communities. In laboratory incubation studies, an increase in salinity might directly limit the adaptability of microorganisms and simultaneously reduce soil respiration^52^, which could reduce oxygen consumption and increase the DO in the overlying water, thereby inhibiting the synthesis of nitrous oxide reductase^53^ and decreasing the abundance of *nosZ* gene^30^. Moreover, the increase of DO in the overlying water might inhibit the activity of anaerobic denitrifying bacteria^54^. Meanwhile, soil microbial populations usually adapt to freshwater or low salt environments, and the decrease in the gene abundances of denitrifying bacteria is potentially due to osmotic stress of microbial cells caused by elevated salinity levels^55^, which interrupts cellular function, growth and even cell lysis^56^. In addition, salinity has a direct inhibitory effect on denitrifying bacteria, with Cl^-^ toxicity suggested as the possible reason^23^. Furthermore, the reduced activity of nitrifying bacteria and nitrifying enzymes by the chloride (Cl^-^) in saline environments further inhibits nitrification^22,23^ and decreases the substrate for denitrification.

It has been reported that the optimal pH range for denitrifiers is 7–9^57^; values beyond this range may hinder the denitrification process and lead to the accumulation of intermediates: NO_2_^-^, NO_2_ and N_2_O^57^. However, the pH of the sediment in the saline-alkali environment exceeding the suitable range of denitrifiers. High pH in the saline-alkali soils further decreased denitrifying gene abundance and denitrification rates (Figs. 1a, 2). Furthermore, great quantities of denitrifying bacteria attach to overlying water–sediment interfaces^58^, and the high pH may inhibit microbial processing of N by reducing the efficiency of enzymatic attacks^59^. The denitrification rates were significantly and positively correlated with NO_3_^–^N in the overlying water and sediment in the saline environment (Fig. 3a; Fig. S2). However, there was no significant correlation between the denitrification rates and sediment NO_3_^–^N in the saline-alkali environment (Fig. 3b; Fig. S3), although the NO_3_^–^N concentration of the sediment in the saline-alkali environment was higher than that in the saline environment. This indicated that the utilization of NO_3_^–^N in the sediment by denitrifiers may be inhibited in saline-alkali environments because the high pH possibly affects microbial access to N resources^59^. The NO_3_^–^N in the overlying water directly provided a substrate for denitrifiers and may have played a more vital role in the denitrification rates than did the NO_3_^–^N of the sediment in the saline-alkali environments (Fig. 6)^22^. This study suggested that the denitrification process became inactive or inhibited in a saline-alkali environment with a high pH, which is in accordance with Anderson et al.^59^. As confirmed by the present study, salinity and high pH had a great impact on N cycling by affecting the abundance of functional genes in the short term.

### Implications for non-point N pollution management

Efforts to reduce non-point source N pollution from watersheds to coastal water are underway around the world^60,61^, and decreasing the output load of non-point source N pollution from ditches to receiving water bodies is an important way to achieve this goal. Improving the removal efficiency of Nr in ditches can effectively reduce the output load of non-point source N pollution. The N removal efficiency in the short term is related to the original N content and denitrification rate. Although the high original N concentration promoted the denitrification rate (Fig. 1a), the N removal efficiency in the short term of the treatments with low urea applications was higher than that of the treatments with high urea applications under the same salinity (Fig. 1b). In farmland drainage ditches with more exogenous N input, although the denitrification rate is high in the short term, it still takes a long time to eliminate non-point source N through denitrification. Therefore, the denitrification time can be prolonged by increasing the hydraulic retention time of the ditches to achieve a high N removal efficiency^62^. However, the relationship between the time required to fully remove non-point source N by denitrification and the initial N concentration in saline-alkali soils needs to be further studied.

Non-point source N output load management plays a critical role in achieving water quality goals^63^. At this stage, China’s agricultural non-point source N pollution management technology mainly focuses on three fields, namely, pollution source reduction, pollutant migration interception, and nutrient recycling^64^. Among them, pollutant migration interception refers to the interception, removal and utilization of non-point source N pollutants by physical, chemical or biological means during their migration into and accumulation in rivers and lakes in order to reduce the pollutant load of ditches and the concentration of pollutants in the lakes^65^. The denitrification process in the ditches is an effective way to intercept the migration of Nr into the rivers. A full understanding of the impact of saline-alkali environments on denitrification rates is conducive to formulating reasonable pollutant migration interception strategies for ditch pollutants and effectively improving the N removal efficiency of ditches in coastal areas. In coastal areas, the high salinity and pH of the soil in ditches inhibit the denitrification rates and hinder the reduction of nitrate, thus reducing the interception by ditches on pollutant migration. The combination between non-point source N pollution control and basin ditch management is highly recommended^59^. In the daily management of ditches, corresponding measures such as chemical improvements and irrigation leaching technologies should be adopted to reduce the salt and alkali contents of ditches and improve denitrification potential. In addition, the removal effect of denitrification on reactive nitrogen in ditches can be further increased by prolonging the hydraulic retention time of ditches. Furthermore, when estimating non-point source N pollution loads, the inhibition effects of saline-alkali environments on denitrification in ditches should be considered.

## Conclusion

Based on flooded incubation experiments of farmland ditches under various salt and salt-alkali gradients, we examined the changes in denitrification rates and denitrifying gene abundance in saline and saline-alkali environments. With an increase in the salt gradient from 1‰ to 3‰, 8‰, and 15‰, the denitrification rates significantly decreased by 23.83–50.08%, 20.64–57.31% and 6.12–54.61% under the addition of 0.05‰, 0.10‰ and 0.15‰ urea conditions, respectively. The inhibition of denitrification rates in a saline-alkali environment with a high pH was stronger than that in a saline environment. With the increase in the salt-alkali gradient from 0.5‰ to 1‰, 3‰, and 8‰, the denitrification rates significantly decreased by 44.57–59.44%, 54.88–65.19% and 57.84–63.24% under the addition of 0.05‰, 0.10‰ and 0.15‰ urea conditions, respectively. The abundance of nitrite reductase genes (*nirK* and *nirS*) slightly decreased under a low salt gradient but decreased significantly under high salt conditions, and the abundance of the nitrous oxide reductase gene (*nosZ*) sharply decreased in the saline environment. Compared with the saline environment, the saline-alkali environment decreased the abundance of denitrifying genes more significantly. In addition, the increase in N concentration could limit the reduction in denitrifying gene abundance. The salinity, DO in the overlying water, N concentration and denitrifying gene abundance were key determinants of the denitrification rate. In the saline environment, the salinity and DO were the main limiting factors of the denitrification rate, and in the saline-alkali environment, high pH was also a determinant. Salinity and high pH decreased the denitrification rates by both inhibiting the abundance of denitrifying genes *nirK*, *nirS*, and *nosZ* and reducing the substrate availability for denitrification process. In addition, the decrease of substrate availability would also affect the abundance of denitrifying genes.

## Supplementary Information

Supplementary Information.
