# Supplementary material for: Salinity and high pH reduce denitrification rates by inhibiting denitrifying gene abundance in a saline-alkali soil
Source: Sci Rep. 2023 Feb 7;13:2155. doi: 10.1038/s41598-023-29311-7 (PMC9905596; doi:10.1038/s41598-023-29311-7)
Supplement: Supplementary file 1 — Supplementary Information. [file 41598_2023_29311_MOESM1_ESM.docx]

**Supplementary information**

## **Text S1.** The calculation of Nitrogen removal efficiency.

Nitrogen removal efficiency (RE) is the ratio of the nitrogen removed by denitrification within 24 hours to the nitrogen added in the incubator. Nitrogen removal efficiency within 24 hours was determined by the following Equation during incubation.

$$RE={(6.72\times{10}^{-4}\cdot DNF\cdot S)}/{\left( m_{s}\cdot a_{N} \right)\times100\%}$$

Where *DNF* indicates the denitrification rate (µmol m^-2^ h^-1^) after 24 h of fertilization, which is considered as the average denitrification rate within 24 hours; *S* (m^-2^) indicates the area of the incubator; *m_s_* (kg) indicates the mass of soil in the incubator; *a_N_* (g N/kg) indicates the nitrogen concentration in the incubator under different urea addition levels; 6.72×10^-4^ is the conversion factor.

## **Text S2.**

For PCR amplification of all functional genes, a microfluidics Fluidigm Gene Expression chip was used to quantify all genes simultaneously. The thermocycler program was 95 °C for 10 min followed by 14 cycles of 95 °C for 15 s and 58 °C for 4 min. A 5-μL mixture was then prepared with a final concentration of 1X SsoFast EvaGreen Supermix with Low Rox (Bio-Rad Laboratories, Hercules, CA), 1X DNA Binding Dye Sample Loading Reagent (Fluidigm, San Francisco, CA), and 2.25 μl pre-amplified product. A separate master mix was prepared with a final concentration of 1X Assay Loading Reagent (Fluidigm, San Francisco, CA), 0.5X DNA Suspension Buffer (Teknova, Hollister CA), and 50 μM of each forward and reverse primer. Each 5-μL mixture containing product was mixed with 5 μL of master mix and loaded onto a 96.96 Fluidigm Gene Expression chip. Fluidigm amplification was performed according to the following program: 70 °C for 40 min, 58 °C for 30 s, 95 °C for 1 min followed by 30 cycles of 96 °C for 5 s, 58 °C for 20 s, and followed by dissociation curve. Standards for each gene were prepared from sample-derived amplicons from a mixture of soils that were quantified and serially diluted prior to analysis on the Fluidigm system (Schmidt et al., 2019). Standard curves were obtained using serial dilutions of linearized plasmids (pGEM-T, Promega) (from 10^5^–10^9^ for denitrifiers) containing cloned *nirK*, *nirS* and *nosZ* Clade I genes amplified from the soil used in this study. Controls without templates resulted in an undetectable signal in all runs, and inhibitory effects were not detected at the chosen dilution. Each sample was established triple holes. If the difference between boles is less than 1Ct, it shall be recorded as effective data, and the average value been taken for the next analysis. If the difference between holes is greater than 1Ct, the test shall be repeated once.

**Reference**

Schmidt, J.E., Kent, A.D., Brisson, V.L., Gaudin, A.C.M. 2019. Agricultural management and plant selection interactively affect rhizosphere microbial community structure and nitrogen cycling. Microbiome, 7(1).


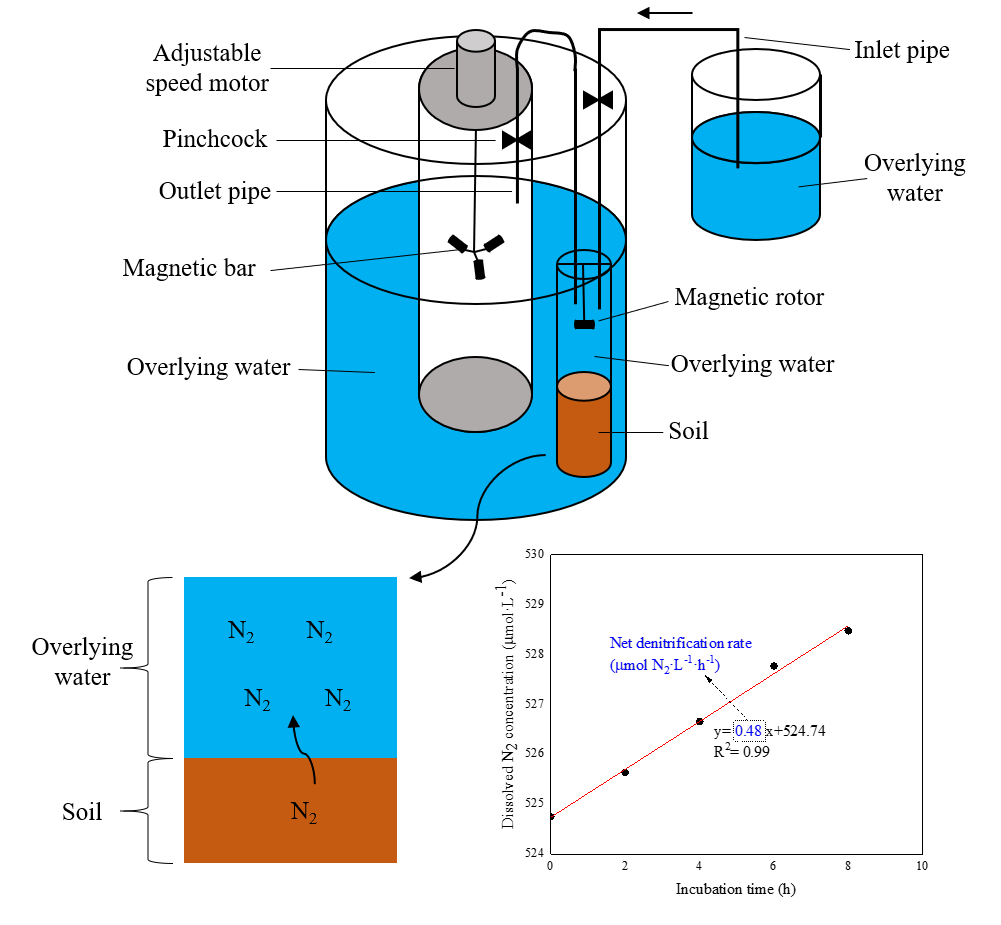


## **Fig. S1** Conceptual diagram of in-situ flow-through incubation device and calculation diagram of net denitrification rate in the water-soil interface (figure quoted from Pan et al. 2021).

Generally, undisturbed soil samplers (PVC, inner diameter 8 cm, outer diameter 9 cm, height 30 cm) were gently immersed in the samples to collect soil cores from the incubators, and then, the bottoms were sealed with rubber stoppers. The overlying water solutions of each corresponding treatment were collected and slowly added into the soil cores and flow-through incubation device. The soil cores were placed vertically in the incubation device simulating the in-situ flooding environment and the water level was approximately 4-5 cm above the samplers, and then equilibrated overnight with gentle aeration from an aquarium air pump. After 8-12 h of immersion to achieve a stable state, the covers of the soil samples were gently screwed on and adjusted until there were no bubbles in the samplers, and then the samplers were connected to inlet and outlet pipes. The inlet pipe was connected to replenishment liquid above the incubator. When a sample was taken, the replenishment liquid automatically replenished the water sample flowing out of the incubator to ensure that there were no bubbles throughout the experiment. Three water samples from each core container were collected at 2-h intervals during the 8-h incubation (collected at 0, 2, 4, 6 and 8 h) and preserved in Exetainer vials (Labco Limited, Buckinghamshire, UK) with 20 μL of a saturated HgCl_2_ solution. Changes in the N_2_ concentration were determined by the following Equation for a given temperature and salinity during incubation.

$$F={[(\left( {N_{2}}/{A_{r}} \right)_{outlet}-\left( {N_{2}}/{A_{r}} \right)_{inlet})\times A_{r}\times V]}/A$$

where F indicates the N_2_ flux (µmol m^-2^ h^-1^) of the soils during the denitrification process; A_r_ indicates the argon concentration (µmol L^-1^); N_2_/A_r_ indicates the ratio of N_2_ concentration to argon concentration; V indicates the flow rate (L h^-1^); and A indicates the surface area of the soils (m^2^). The net N_2_ flux was calculated for each sample by extrapolating the rate of N_2_ change from a five-point linear regression over the incubation time.

**Reference**

Pan, Y.C., She, D.L., Shi, Z.Q., Chen, X.Y., Xia, Y.Q. 2021. Do biochar and polyacrylamide have synergistic effect on net denitrification and ammonia volatilization in saline soils? Environ. Sci. Pollut. R. 28(42), 59974-59987.

## **Fig. S2** Bivariate linear relationship between the explanatory variables and denitrification rate under single salt condition.

## **Fig. S3** Bivariate linear relationship between the explanatory variables and denitrification rate under salt-alkali condition.

## **Table S1** The summary of results of environmental factors

| Treatment | | Overlying water | | | | | | Sediment | | | | |
| --- | --- | --- | --- | --- | --- | --- | --- | --- | --- | --- | --- | --- |
| Urea level | Salt/salt-alkali gradient | NH_4_^+^-N  (mg N/L) | NO_3_^-^-N  (mg N/L) | DO  (mg/L) | EC  (mS/cm) | Na^+^  (g/L) | pH | NH_4_^+^-N  (mg N/kg) | NO_3_^-^-N  (mg N/kg) | EC  (μS/cm) | Na^+^  (g/kg) | pH |
| N1 | CK | 2.481±1.09cd | 0.156±0.03a | 8.06±0.14bcd | 2.18±0.08e | 0.28±0.02e | 7.35±0.13d | 1.433±0.37a | 0.468±0.08c | 520.33±21.08e | 0.132±0.01f | 8.25±0.07f |
|  | S1 | 2.281±1.75cd | 0.138±0.09ab | 8.10±0.14bcd | 3.84±1.19cd | 0.31±0.02e | 7.72±0.16c | 1.209±0.28ab | 0.427±0.05c | 605.00±47.02e | 0.180±0.03e | 8.32±0.01ef |
|  | S2 | 1.078±0.14d | 0.115±0.04abc | 8.22±0.15abc | 4.58±1.26c | 0.41±0.03d | 7.90±0.27bc | 0.665±0.35bc | 0.413±0.05c | 1016.33±29.49c | 0.327±0.00d | 8.39±0.08de |
|  | S3 | 0.922±0.39d | 0.094±0.06abc | 8.34±0.15ab | 9.78±0.46b | 0.75±0.01b | 7.94±0.11bc | 0.514±0.16c | 0.418±0.04c | 1920.00±282.84b | 0.557±0.04b | 8.26±0.10f |
|  | S4 | 0.773±0.26d | 0.073±0.01abc | 8.52±0.27a | 16.69±0.65a | 0.95±0.06a | 7.95±0.04bc | 0.481±0.05c | 0.417±0.15c | 2846.67±253.16a | 0.725±0.02a | 8.40±0.02de |
|  | A1 | 4.710±0.95a | 0.086±0.02abc | 7.82±0.11d | 2.42±0.04de | 0.30±0.02e | 7.96±0.04bc | 1.128±0.45abc | 1.821±0.25a | 554.67±14.43e | 0.177±0.00e | 8.45±0.02d |
|  | A2 | 4.450±1.00ab | 0.053±0.00bc | 7.97±0.12cd | 2.65±0.16de | 0.31±0.03e | 8.00±0.07bc | 0.931±0.31abc | 1.384±0.48b | 584.33±17.79e | 0.181±0.01e | 8.59±0.04c |
|  | A3 | 3.410±0.34abc | 0.043±0.00bc | 8.12±0.04bcd | 2.88±0.22de | 0.39±0.04d | 8.12±0.08b | 0.584±0.31bc | 0.709±0.07c | 714.33±10.87de | 0.302±0.01d | 9.44±0.02b |
|  | A4 | 2.653±0.08bcd | 0.033±0.01c | 8.33±0.10ab | 3.59±0.16cde | 0.65±0.06c | 8.52±0.10a | 0.502±0.08c | 0.375±0.02c | 931.67±8.73cd | 0.467±0.02c | 9.91±0.02a |
| N2 | CK | 4.393±1.86a | 2.751±1.00a | 7.94±0.19bc | 2.13±0.15d | 0.28±0.04e | 7.95±0.32bc | 3.493±0.89a | 0.672±0.18b | 681.33±171.74ef | 0.183±0.06e | 8.22±0.06e |
|  | S1 | 3.407±3.00b | 0.827±0.73b | 8.07±0.14ab | 3.27±0.29d | 0.37±0.01d | 7.81±0.08bc | 2.137±0.48abcd | 0.698±0.08b | 928.00±161.04de | 0.268±0.06d | 8.26±0.12e |
|  | S2 | 2.997±0.29b | 0.113±0.08c | 8.08±0.14ab | 6.23±1.79c | 0.47±0.03c | 7.96±0.05bc | 1.720±0.32bcd | 0.531±0.07b | 1195.33±84.84c | 0.367±0.03c | 8.30±0.05e |
|  | S3 | 2.680±0.99b | 0.108±0.04cd | 8.29±0.18a | 10.76±0.93b | 0.73±0.05b | 7.71±0.03c | 1.616±0.58bcd | 0.539±0.06b | 1766.00±111.45b | 0.520±0.02b | 8.33±0.02de |
|  | S4 | 2.453±0.30b | 0.086±0.05d | 8.37±0.16a | 17.32±1.30a | 0.95±0.01a | 8.01±0.03b | 0.892±0.30cd | 0.458±0.14b | 2733.33±168.59a | 0.703±0.02a | 8.29±0.07e |
|  | A1 | 7.183±2.31a | 0.142±0.03c | 7.75±0.11c | 2.43±0.21d | 0.27±0.03e | 8.01±0.03b | 2.825±1.31ab | 2.629±1.07a | 564.00±31.63f | 0.167±0.02e | 8.44±0.04cd |
|  | A2 | 5.100±0.19ab | 0.076±0.01d | 7.91±0.14bc | 2.50±0.14d | 0.36±0.04d | 8.03±0.00b | 2.510±0.82abc | 2.603±1.38a | 577.33±38.39f | 0.185±0.01e | 8.51±0.02c |
|  | A3 | 5.360±1.13ab | 0.053±0.01d | 8.10±0.05ab | 2.70±0.18d | 0.45±0.04c | 8.08±0.04b | 1.937±0.95abc | 1.513±0.73ab | 829.00±89.83de | 0.339±0.02c | 9.41±0.05b |
|  | A4 | 4.643±1.14ab | 0.054±0.02d | 8.22±0.04ab | 3.49±0.19d | 0.70±0.03b | 8.60±0.15a | 0.673±0.11d | 0.372±0.01b | 989.67±30.47cd | 0.502±0.01b | 9.95±0.05a |

## **Continued Table S1** The summary of results of environmental factors

| Treatment | | Overlying water | | | | | | Sediment | | | | |
| --- | --- | --- | --- | --- | --- | --- | --- | --- | --- | --- | --- | --- |
| Urea level | Salt/salt-alkali gradient | NH_4_^+^-N  (mg N/L) | NO_3_^-^-N  (mg N/L) | DO  (mg/L) | EC  (mS/cm) | Na^+^  (g/L) | pH | NH_4_^+^-N  (mg N/kg) | NO_3_^-^-N  (mg N/kg) | EC  (μS/cm) | Na^+^  (g/kg) | pH |
| N3 | CK | 4.687±2.69cd | 3.814±0.25a | 7.61±0.38bc | 2.40±0.17e | 0.24±0.02e | 7.73±0.02d | 5.947±0.65ab | 0.815±0.12cd | 615.67±108.60ef | 0.167±0.04fg | 8.23±0.04e |
|  | S1 | 4.400±0.75cd | 3.511±1.14a | 7.84±0.20abc | 3.18±0.26de | 0.33±0.03de | 7.87±0.07cd | 3.980±1.25bcd | 0.779±0.20cd | 779.67±144.97cde | 0.232±0.05ef | 8.35±0.04de |
|  | S2 | 3.793±0.71d | 2.099±0.83b | 7.90±0.20abc | 4.83±0.45c | 0.42±0.05cd | 7.88±0.07cd | 3.323±1.39bcd | 0.706±0.09cd | 960.33±145.83c | 0.292±0.03de | 8.38±0.04d |
|  | S3 | 3.287±0.63d | 1.075±1.12bc | 8.01±0.13ab | 10.08±1.36b | 0.70±0.05b | 7.90±0.03cd | 2.927±0.84cd | 0.554±0.13d | 1793.67±121.16b | 0.522±0.03b | 8.32±0.04de |
|  | S4 | 3.030±0.43d | 0.614±0.46c | 8.17±0.17a | 16.57±0.65a | 0.94±0.02a | 7.89±0.02cd | 2.120±0.45d | 0.520±0.08d | 2913.33±87.31a | 0.727±0.03a | 8.36±0.09de |
|  | A1 | 11.107±1.43a | 0.152±0.01c | 7.51±0.17c | 2.36±0.10e | 0.27±0.05e | 8.00±0.06bc | 8.427±2.15a | 4.067±1.09a | 541.67±30.40f | 0.152±0.02g | 8.39±0.04d |
|  | A2 | 10.410±0.50ab | 0.141±0.03c | 7.68±0.12bc | 2.56±0.12e | 0.29±0.04e | 8.01±0.03bc | 5.313±1.63bc | 3.550±1.22ab | 561.00±31.89f | 0.178±0.01fg | 8.56±0.04c |
|  | A3 | 7.770±1.41b | 0.065±0.01c | 8.00±0.10ab | 2.78±0.05de | 0.46±0.06c | 8.12±0.18b | 2.237±0.53d | 2.214±1.18bc | 710.00±41.38def | 0.300±0.02d | 9.36±0.12b |
|  | A4 | 7.353±2.05bc | 0.048±0.00c | 8.20±0.06a | 3.95±0.05cd | 0.62±0.09b | 8.66±0.05a | 2.553±0.54d | 0.386±0.02d | 906.33±20.98cd | 0.456±0.01c | 9.89±0.08a |

Means ± standard errors (n=3).

## **Table S2** Overview of the PLSR model of the denitrification rate in a saline environment.

| Component | R^2^(X) | R^2^(X)_cum_ | R^2^(Y) | R^2^(Y) _cum_ | Q_cum_^2^ |
| --- | --- | --- | --- | --- | --- |
| 1 | 0.507 | 0.507 | 0.834 | 0.834 | 0.824 |
| 2 | 0.151 | 0.658 | 0.068 | 0.902 | 0.887 |

## **Table S3** Overview of the PLSR model of the denitrification rate in a saline-alkali environment.

| Component | R^2^(X) | R^2^(X)_cum_ | R^2^(Y) | R^2^(Y) _cum_ | Q_cum_^2^ |
| --- | --- | --- | --- | --- | --- |
| 1 | 0.530 | 0.530 | 0.677 | 0.677 | 0.646 |
| 2 | 0.183 | 0.713 | 0.241 | 0.918 | 0.894 |
